# Triple threat: how diabetes results in worsened bacterial infections

**DOI:** 10.1128/iai.00509-23

**Published:** 2024-03-25

**Authors:** Benjamin P. Darwitz, Christopher J. Genito, Lance R. Thurlow

**Affiliations:** 1 Department of Microbiology and Immunology, University of North Carolina at Chapel Hill School of Medicine, Chapel Hill, North Carolina, USA; 2 Division of Oral and Craniofacial Health Sciences, University of North Carolina at Chapel Hill Adams School of Dentistry, Chapel Hill, North Carolina, USA; University of Pittsburgh, Pittsburgh, Pennsylvania, USA

**Keywords:** diabetes, bacterial infection, antibiotic resistance, hyperglycemia, *Staphylococcus aureus*, *Pseudomonas aeruginosa*, inflammation

## Abstract

Diabetes mellitus, characterized by impaired insulin signaling, is associated with increased incidence and severity of infections. Various diabetes-related complications contribute to exacerbated bacterial infections, including hyperglycemia, innate immune cell dysfunction, and infection with antibiotic-resistant bacterial strains. One defining symptom of diabetes is hyperglycemia, resulting in elevated blood and tissue glucose concentrations. Glucose is the preferred carbon source of several bacterial pathogens, and hyperglycemia escalates bacterial growth and virulence. Hyperglycemia promotes specific mechanisms of bacterial virulence known to contribute to infection chronicity, including tissue adherence and biofilm formation. Foot infections are a significant source of morbidity in individuals with diabetes and consist of biofilm-associated polymicrobial communities. Bacteria perform complex interspecies behaviors conducive to their growth and virulence within biofilms, including metabolic cross-feeding and altered phenotypes more tolerant to antibiotic therapeutics. Moreover, the metabolic dysfunction caused by diabetes compromises immune cell function, resulting in immune suppression. Impaired insulin signaling induces aberrations in phagocytic cells, which are crucial mediators for controlling and resolving bacterial infections. These aberrancies encompass altered cytokine profiles, the migratory and chemotactic mechanisms of neutrophils, and the metabolic reprogramming required for the oxidative burst and subsequent generation of bactericidal free radicals. Furthermore, the immune suppression caused by diabetes and the polymicrobial nature of the diabetic infection microenvironment may promote the emergence of novel strains of multidrug-resistant bacterial pathogens. This review focuses on the “triple threat” linked to worsened bacterial infections in individuals with diabetes: (i) altered nutritional availability in diabetic tissues, (ii) diabetes-associated immune suppression, and (iii) antibiotic treatment failure.

## INTRODUCTION

Diabetes mellitus presents a significant global health crisis, with projections estimating that 783 million individuals will be diagnosed with diabetes worldwide by 2045 (1). One substantial health complication associated with diabetes is an increased vulnerability to bacterial infections, marked by higher frequency and severity compared with individuals without diabetes (2–8). Thus, understanding the intricate mechanisms underlying diabetes-associated complications and how they alter bacterial infection dynamics is fundamental for informing future research.

Diabetes is principally characterized by impaired insulin signaling cascades resulting from insufficient insulin production [type 1 diabetes (T1D)] or insulin resistance (type 2 diabetes, T2D) (9–12). T1D arises from autoimmune-mediated destruction of pancreatic islet β-cells responsible for the production and secretion of insulin (11–13). Insulin resistance and T2D develop from a complex interplay of factors, including chronic low-grade inflammation and hyperinsulinemia, leading to the dysfunction of insulin receptor proteins expressed on cell surfaces (14–16). T2D accounts for approximately 90% of all diabetes cases, and the primary risk factors contributing to its development include obesity, sedentary lifestyles, high-caloric diets, and population aging (9–12). A further complication that arises in obese individuals with T1D is the development of insulin resistance, leading to a state known colloquially as “double diabetes” (17, 18). Functional insulin signaling maintains essential cellular processes, including glucose uptake and metabolism, biomacromolecule synthesis, and cell proliferation (19–21). As such, the repercussions of diabetes extend beyond metabolic disturbances, contributing to numerous health complications, including vascular disease, neuropathy, myopathy, and dysregulated immune responses (15, 22, 23). Although each of these factors adds to the heightened morbidity and mortality associated with diabetes, the mechanisms contributing to the increased susceptibility of individuals with diabetes to bacterial infections are complex and multifactorial.

The altered nutrient composition of the diabetic infection microenvironment contributes to increased bacterial burden and virulence during infections. Hyperglycemia, the hallmark symptom of uncontrolled diabetes, is a pivotal factor in worsening bacterial infections by serving as an ideal growth environment for pathogens (4–6, 24–26). Individuals with poorly managed glycemic control exhibit elevated glucose concentrations in tissues and organs throughout the body (27–29). Glucose is the preferred carbon source of numerous bacterial pathogens, including staphylococcal, streptococcal, and enterococcal species, which use glucose to fuel their growth and virulence potential (30–32). In line with these observations, individuals with diabetes exhibit a greater incidence of skin and soft tissue infections (SSTIs) arising from Gram-positive cocci species than non-diabetic individuals (33–35). SSTIs pose a significant burden on patients with diabetes, leading to complications such as gangrene, osteomyelitis, bacteremia, and sepsis (36–38). Diabetic SSTIs can range from mild skin infections to severe necrotizing fasciitis and most frequently manifest on the lower extremities, known as diabetic foot infections (DFIs)(35–38). DFIs are particularly problematic, necessitating frequent hospitalizations and serving as the most common precipitating event to lower extremity amputations (39–41). Several bacterial pathogens exhibit increased growth and virulence potential during infection of hyperglycemic tissues, including *Staphylococcus aureus*, *Enterococcus faecalis*, and *Streptococcus agalactiae* [group B streptococcus (GBS)] (30–33). Left untreated, infecting bacteria in DFIs can penetrate further into the tissue and cause the development of secondary infections, including osteomyelitis, which is the second leading cause of amputation in the United States (39–42).

Concomitant with the altered nutrient environment in diabetic tissues enhancing bacterial growth and virulence, diabetes is further associated with compromised immune responses (2, 4, 15, 23). The immune suppression associated with diabetes manifests at various levels, ranging from diminished immune cell recruitment at infection sites to alterations in cytokine and chemokine profiles (43–47). Furthermore, diabetes impairs the metabolic mechanisms essential for free radical generation by professional phagocytes, including macrophages and neutrophils (48). The diminished phagocytic and antibacterial capabilities of professional phagocytes in diabetic infections are associated with increased infection rates by facultative intracellular bacterial pathogens (49). Notably, individuals with diabetes exhibit an increased incidence of infection by *Mycobacterium tuberculosis* (mTb) and *Burkholderia pseudomallei*, the causative agents of tuberculosis and melioidosis, respectively (25, 49). Compared with non-diabetic individuals, those with diabetes experience a threefold higher incidence of infection by mTb and a fivefold increased rate of mortality associated with tuberculosis (49). Furthermore, individuals with diabetes exhibit an astounding 13-fold increased rate of infection by *B. pseudomallei*, which can be lethal despite antibiotic intervention (49–51).

The heightened prevalence and severity of infections in individuals with diabetes underscore the need for effective treatments. Increased rates of antibiotic treatment failure are observed in patients with diabetes due to heightened bacterial tolerance to antibiotic compounds (52, 53). Furthermore, evidence from several studies suggests that the diabetic infection microenvironment fosters conditions conducive to the emergence of antibiotic-resistant bacterial strains (54, 55). This alarming phenomenon has important implications regarding the increased incidence of diabetes and how it may contribute to the global health threat of antimicrobial drug resistance and the emergence of multidrug-resistant pathogens. Altogether, the combined impact of altered nutritional, immunological, and therapeutic functions in individuals with diabetes collectively promotes an increased frequency and severity of bacterial infections. This review aims to summarize crucial aspects of diabetes-associated bacterial infections, including how differences in the nutritional composition of diabetic infection microenvironment influence bacterial metabolism and virulence, the mechanisms underlying diabetic immune suppression, and how diabetes contributes to the emergence of antibiotic-resistant bacterial strains.

## BACTERIAL METABOLISM AND THE DIABETIC INFECTION MICROENVIRONMENT

Within the infection microenvironment, the host and infectious bacteria compete for the limited pool of nutrients, including trace minerals, carbohydrates, and amino acids (56–58). The host employs several mechanisms to sequester nutrients from infiltrating bacteria to limit bacterial growth and enhance the bactericidal activity of innate immune cells (57–60). Traditionally, “nutritional immunity” refers to the process by which the host limits pathogen access to trace elements essential for bacterial growth and resistance to the immune response, such as iron, zinc, and manganese (57, 61). However, host sequestration of carbon sources, such as glucose, is essential for limiting bacterial growth and virulence potential (59, 61). Individuals with unmanaged diabetes exhibit vastly altered infection microenvironments that contain elevated concentrations of limiting nutrients readily consumed by pathogenic bacteria (4–6). Here, we will discuss how the altered nutritional environment in diabetic infections contributes to worsened bacterial infections.

Chronic hyperglycemia is a hallmark symptom of inadequately controlled diabetes (28, 29, 62). Extended periods of insulin deficiency can progress into acute metabolic syndromes characterized by elevated blood glucose concentrations, including diabetic ketoacidosis (DKA) and hyperosmolar hyperglycemic state (HHS)(62). DKA primarily afflicts individuals with T1D or ketosis-prone T2D and manifests as a consequence of relative or absolute insulin deficiency (63–65). DKA results in elevated blood glucose levels (>200 mg/dL) from increased gluconeogenesis, enhanced glycogenolysis, and impaired glucose utilization (64, 65). Conversely, HHS without ketoacidosis is frequently associated with T2D (28, 29). HHS is characterized by extremely high blood glucose concentrations (>600 mg/dL), dehydration, and elevated blood osmolarity (66). Persistent elevation of blood glucose levels triggers alterations in host physiological and immunoregulatory functions and is observed to correlate with adverse clinical outcomes in diabetic patients with bacterial infections (43–47).

Elevated glucose concentrations in tissues create microenvironments conducive to bacterial colonization and proliferation (4–6, 24–26, 67). Some bacterial pathogens undergo specific adaptations that enhance their virulence during growth in hyperglycemic conditions (58, 68, 69). Hyperglycemic states can influence the pathogenicity and virulence of some bacterial pathogens in the context of diabetic SSTIs, which range from non-invasive cellulitis to severe abscesses and necrotizing fasciitis, particularly on the lower extremities (29, 35, 38). Although superficial skin infections are typically caused by a singular pathogenic organism, more chronic and severe wounds tend to be polymicrobial (38, 70, 71). The heightened frequency of diabetes-associated skin infections is largely attributed to the increased risk of developing open sores or wounds on the lower extremities that are recalcitrant to healing, known as diabetic foot ulcers (72–74). Approximately 19%–34% of individuals with diabetes will develop a diabetic foot ulcer at some point in their lives, and roughly 50%–60% of these ulcers will result in DFIs (72, 73). Notably, 15%–20% of individuals who develop a DFI will require amputation to adequately control infection, which is associated with a marked decrease in patient life expectancy within the following 2 years (73, 75). Numerous bacterial pathogens are frequently isolated from diabetic skin infections, including several coagulase-negative staphylococci, enterococci, streptococci, clostridia, and pseudomonades (35, 38). However, *S. aureus* is one of the most frequently isolated bacterial pathogens in diabetic wounds (33, 75, 76).

*S. aureus*, including methicillin-resistant *S. aureus* (MRSA), is the most prevalent bacterial pathogen in DFIs worldwide, even when accounting for differences between high-income countries (HIC) and upper-middle and lower-middle income countries (U/LMIC)(33). Glucose is the preferred carbon source of *S. aureus*, which exhibits enhanced virulence potential in diabetic infections (31, 58, 77). *S. aureus* encodes four dedicated glucose transporters to maximize glucose uptake from the environment (31, 58). In hyperglycemic conditions, *S. aureus* utilizes glycolytic and fermentative metabolic pathways to maximize carbon catabolism and the resulting ATP production in a redox-balanced manner, known as overflow metabolism (31, 77, 78). During overflow metabolism, *S. aureus* is capable of catabolizing pyruvate concentrations that exceed the flux of the tricarboxylic acid (TCA) cycle by simultaneously fluxing pyruvate through fermentative pathways (78, 79). The expanded glycolytic capacity of *S. aureus* allows it to resist the bactericidal effects of nitric oxide (NO•) generated by innate immune cells during phagocytosis when glucose is abundant in the environment (80). Additionally, *S. aureus* relies on elevated concentrations of intracellular ATP to activate the accessory gene regulator (Agr) quorum sensing system, which is one of the central systems that regulates *S. aureus* virulence via the small regulatory RNA, RNAIII (58, 77, 81). RNAIII controls the production of numerous *S. aureus* virulence factors, including toxins and proteases that can degrade host tissues and phenol-soluble modulins and leukocidins that lyse host immune cells (81, 82). Although multiple studies demonstrate that *S. aureus* requires the Agr system to establish successful infection in various animal models (58, 83–85), Tuchscherr et al. previously demonstrated that clinical isolates of *S. aureus* recovered from hospitalized patients with diabetes exhibited reduced virulence as measured by hemolytic capacity, degree of invasiveness, and cytotoxicity to human umbilical vein endothelial cells (86). Furthermore, these low-virulence *S. aureus* strains can establish invasive infection in diabetic mice, suggesting that the diabetic infection microenvironment favors *S. aureus* growth regardless of its virulence potential (86). Nevertheless, previous animal model studies demonstrate that laboratory *S. aureus* strains exhibit greater RNAIII expression and virulence factor production during osteomyelitis and SSTI infections in diabetic mice (58, 68). These findings provide evidence that *S. aureus* exhibits enhanced virulence potential during diabetic infections regardless of strain-specific basal virulence activity.

In addition to *S. aureus*, *Enterococcus* spp., including *E. faecalis*, are frequently isolated from diabetic skin wounds, particularly DFIs (76, 87–89). *E. faecalis* preferentially consumes carbohydrates, mainly glucose and maltose, to fuel its growth and biofilm formation (90). *E. faecalis* virulence is regulated by a quorum sensing system, the *E. faecalis* system regulator (Fsr), which shares significant homology with the *S. aureus* Agr quorum sensing system (91, 92). The Fsr pathway involves activation of the transcription factor FsrA, which induces the expression of gelatinase, a serine protease (SprE), and enterocin O16, which are essential components that drive *E. faecalis* virulence (92, 93). Notably, gelatinase drives biofilm formation through the cleavage of autolysins on the surface of *E. faecalis*, resulting in the release of extracellular DNA that is used as scaffolding for biofilm assembly (93, 94). Biofilm formation is an important virulence determinant of *E. faecalis* by conferring protection from antimicrobial compounds and the host immune response (94–96). *In vitro* studies show that *E. faecalis* exhibits increased Fsr-mediated biofilm production in the presence of glucose (91, 97). However, it is unclear whether *E. faecalis* displays enhanced biofilm-forming activity in hyperglycemic tissues during infection. Furthermore, there is a significant knowledge gap regarding whether hyperglycemia or other changes in the nutritional microenvironment of diabetic tissues directly exacerbate the virulence potential of pathogenic enterococci. Nevertheless, enterococci persist as a significant health challenge for individuals with diabetes, given the potential for vancomycin-resistant enterococci (VRE) to drive the emergence of novel antimicrobial resistance in strains of bacterial pathogens (98–100). This phenomenon will be discussed later in this review.

Beyond *Enterococcus* spp., several *Streptococcus* spp. are frequently isolated from diabetic infection sites (30, 35, 71, 101, 102). Streptococci comprise a diverse group of bacteria known to cause severe infection in individuals with diabetes (24, 67, 71). In particular, people with T1D who display poor long-term glycemic control are predisposed to developing bacteremia caused by beta-hemolytic *Streptococcus* spp., including GBS and group G streptococci (24). GBS is considered a significant pathogen in neonates and pregnant women but can also infect non-pregnant individuals with underlying health conditions (30, 102). Notably, diabetes is the most substantial risk factor associated with non-pregnant individuals who develop GBS infections (103). Like *S. aureus* and *E. faecalis*, GBS preferentially utilizes glucose as a carbon source to fuel its growth and biofilm formation (69, 104). GBS virulence factor production is coordinated by the control of virulence (Cov) two-component system (TCS) CovRS (also called CrsRS) (105, 106). The CovRS TCS comprises the CovS sensor histidine kinase, which senses and responds to extracellular stimuli via phosphorylation of the cognate response regulator, CovR (105–107). Upon activation, CovR represses the production of several GBS virulence factors, including the nuclease NucA, the plasminogen-binding protein PbsP, and the *cyl* operon (43), which is responsible for the hemolytic activity of GBS (108). Although the primary signals that stimulate CovS are currently unclear, previous studies speculate that pH fluctuations elicit a CovS response (106). GBS readily acidifies hyperglycemic environments as a direct result of fermentative glucose metabolic pathways (109), which would initially suggest that GBS exhibits reduced virulence in the hyperglycemic environment of diabetic wounds. However, whether GBS sufficiently acidifies the tissue microenvironment of diabetic wounds to the degree required to activate CovS is currently unclear. Interestingly, Keogh et al. demonstrate that GBS acquires mutations in *covR* in diabetic wound infections but not in normoglycemic wounds (43), suggesting that the diabetic infection microenvironment enhances GBS virulence by serving as a highly mutable environment.

Hyperglycemic individuals with T2D and diabetic individuals with indwelling urinary catheters are also at greater risk for developing UTIs, including infection of the bladder (cystitis) and kidneys (pyelonephritis) (110–113). Glycosuria, the presence of glucose in the urine, occurs in individuals with diabetes (114, 115). Elevated glucose concentrations in the urine serve as an abundant nutrient source for several pathogenic bacteria, including *Escherichia coli*, *Klebsiella pneumoniae*, *E. faecalis*, and GBS (30, 69, 102, 111). However, hyperglycemia can also induce host-specific physiological changes that enhance bacterial pathogenicity. For example, the mechanism by which uropathogenic *E. coli* (UPEC) exhibits enhanced pathogenicity during diabetic UTIs is mediated by alterations in host cell surface proteins (116). UPEC is among the most common causative agents of community-acquired UTIs in non-diabetic and diabetic individuals (116). Previous studies show that UPEC and non-uropathogenic *E. coli* isolates exhibit enhanced growth in glycosuria compared with normal urine (111, 117). UPEC employs an arsenal of virulence factors to establish infection, including adhesins, type 1 pili, lipopolysaccharides (LPS), and flagella (116). An integral virulence mechanism of UPEC during UTIs involves establishing intracellular bacterial communities within bladder epithelial cells while avoiding host clearance mechanisms (118). Type 1 pili serve as the primary adherence appendages of UPEC by binding to mannose oligosaccharides on host cell surfaces (116). Previous studies indicate that poorly managed hyperglycemia and resulting glycosuria can lead to the accumulation of advanced glycation end products (AGEs) on the surface of urothelial cells, which serve as alternative binding receptors for type 1 pili, enhancing UPEC adhesion and subsequent urothelial cell infiltration (119).

Although hyperglycemic conditions within the diabetic infection microenvironment can enhance the growth and virulence potential of *S. aureus*, *Enterococcus* spp., *Streptococcus* spp., and UPEC, it is also necessary to consider how hyperglycemia can modulate the interactions between bacteria during polymicrobial infections. Advanced diabetic skin infections are frequently polymicrobial (35, 75), and studies suggest that polymicrobial diabetic infections necessitate altered treatment therapies and can result in worsened clinical outcomes (71, 120, 121). Although numerous bacterial pathogens and pathobionts are frequently isolated from polymicrobial DFIs, a meta-analysis comprising 112 studies involving microbiological prevalence data in DFIs reveals that several bacterial genera and species are enriched in DFIs in both HIC and U/LMIC, with *S. aureus*, *Pseudomonas* spp., *E. coli*, *Enterococcus* spp., and *Proteus* spp. occurring at a greater than 5% prevalence in both HIC and U/LMIC (33). During multispecies infections, bacteria typically cohabitate within aggregated communities encased in extracellular polymeric substances, known as biofilms (70). Within biofilms, bacteria exhibit complex interspecies relationships that involve various degrees of synergy or antagonism mediated by sharing or hoarding nutrients, production of factors that can enhance or inhibit the growth of other species, and secretion of compounds with protective or bactericidal activities (70). Glucose availability is associated with enhanced biofilm-forming characteristics in several bacteria, including *S. aureus* and *Pseudomonas aeruginosa* (122, 123).

*S. aureus* and *P. aeruginosa* are frequently implicated as the primary infectious agents associated with pulmonary decline in individuals with cystic fibrosis (CF) and are known to primarily co-exist within the CF lung environment in biofilm communities (124, 125). *S. aureus* primarily colonizes the lungs of children and adolescents with CF, whereas *P. aeruginosa* typically emerges as a primary infectious bacteria in adults with CF (81). *P. aeruginosa* secretes several antistaphylococcal factors that are known to inhibit the growth of or otherwise kill *S. aureus* (126). In support of this observation, longitudinal data demonstrates that the emergence of *P. aeruginosa* within the lungs of adults with CF corresponds to a decline in the incidence of CF-associated *S. aureus* infections (127). However, recent work from Fischer et al. contradicts the assumption that *P. aeruginosa* slowly replaces *S. aureus* in the CF lung environment, with evidence showing that *S. aureus* and *P. aeruginosa* both accumulate in the CF lung during co-infection (128).

A common comorbidity associated with CF is the development of CF-related diabetes (CFRD), which is becoming increasingly prevalent in individuals with CF with the advent of more effective treatment therapeutics, ultimately resulting in longer CF patient lifespans (129). Previous studies demonstrate that CFRD is associated with increased incidence of *S. aureus* and *P. aeruginosa* co-infections (130) and increased airway glucose concentrations enhance *S. aureus* and *P. aeruginosa* growth (131). Furthermore, *S. aureus* exhibits reduced growth inhibition by *P. aeruginosa* during subcutaneous catheter-associated co-infections within diabetic mice (132). Altogether, these data suggest that glucose alters the co-infection dynamics between *S. aureus* and *P. aeruginosa* by exacerbating the burden of both species during diabetic infections. *P. aeruginosa* secretes numerous toxins that inhibit the electron transport chain of *S. aureus*, which induces a small colony variant (SCV) phenotype in *S. aureus* (133). SCV *S. aureus* relies solely on fermentative metabolic pathways for ATP generation and balancing redox (134), thereby allowing *S. aureus* to mitigate complete elimination during co-habitation with *P. aeruginosa* (135). Additionally, *S. aureus* fermentation results in the accumulation of acidic by-products, including lactate, which is preferentially consumed by *P. aeruginosa* (134). In hyperglycemic conditions, overflow metabolism allows *S. aureus* to simultaneously generate ATP and balance redox (31, 79, 80), which may contribute to the ability of *S. aureus* to grow in the presence of *P. aeruginosa* in conditions with accessible glucose.

In addition to glycolytic end-products, other metabolic end-products derived from *S. aureus* are known to contribute to interspecies cross-feeding, including heme. *S. aureus* encodes two heme-dependent terminal oxidases that facilitate the transfer of electrons from the ETC to the final electron acceptor oxygen (136). *S. aureus* requires heme for aerobic respiration and utilizes several mechanisms to both exogenously acquire and endogenously synthesize heme (137). Conversely, several pathogenic bacteria cannot synthesize heme and instead rely solely on exogenous heme acquisition systems (138). For example, *E. faecalis* cannot synthesize the heme precursor porphyrin due to its lack of a functional TCA cycle (32, 139). Previous work has shown that *E. faecalis* utilizes *S. aureus*-derived heme during co-culture, which drives *E. faecalis* biofilm formation (139). Similarly, GBS lacks several enzymes crucial for respiration metabolism during mono-culture but can utilize exogenous heme and quinones to perform respiration (140). Furthermore, GBS produces increased levels of NucA during respiration metabolism, which is required for maximal GBS virulence (141). Altogether, these findings suggest that the cross-feeding behaviors of pathogenic bacteria within DFIs may fuel their growth and virulence potential.

Besides glucose, diabetes is further associated with alterations in trace element metabolism (61, 142). Several trace elements, including iron, manganese, zinc, and copper, play crucial roles in maintaining proper cellular function by supporting various physiological processes in humans and bacteria (57). For example, humans and many bacterial species utilize iron to facilitate enzymatic processes, perform respiration, and carry out DNA synthesis and repair (143). Individuals with insulin resistance and T2D demonstrate aberrancies in iron homeostasis marked by increased serum concentrations of ferritin, the primary form of iron storage in the human body (142, 144). Few studies explore whether diabetes and associated aberrations in trace element metabolism influence the severity of bacterial infections. Keogh et al. show that GBS exhibits reduced expression of genes involved with iron, zinc, and manganese transport and increased expression of genes associated with iron export during infection of diabetic wounds relative to GBS isolated from non-diabetic wounds (43). Based on these observations, Keogh et al. hypothesize that diabetic wounds potentially contain heightened concentrations of trace metals, which causes GBS to downregulate genes involved with metal transport to presumably avoid cellular toxicity. However, whether diabetic tissues contain elevated or reduced concentrations of trace elements remains ambiguous. Numerous studies assessing concentrations of trace metals in the serum of individuals with diabetes compared with normoglycemic individuals reveal conflicting results. For example, the concentration of serum zinc in individuals with diabetes ranges from lower (145), similar (146, 147), and higher (148) compared with control groups. Furthermore, individuals with DFIs display altered serum concentrations of copper, zinc, and magnesium compared with uninfected individuals with diabetes (147). Despite inconclusive findings in the current literature concerning differences in trace mineral concentrations within diabetic tissues, it remains a potential contributor to exacerbated bacterial infections in individuals with diabetes, thus warranting further study.

Individuals with insulin resistance and T2D display increased levels of circulating proteins involved with sequestering trace elements from bacteria (149, 150). Specifically, these proteins belong to the S100 protein family and facilitate mechanisms of nutritional immunity (150, 151). The S100 proteins, which encompass calprotectin, calgranulin C, and psoriasin, are calcium-binding proteins that exert an array of immunoregulatory functions and sequester metals from pathogens (150, 151). Calprotectin is the most abundant cytosolic protein within neutrophils, where it participates in calcium signaling pathways (152). Neutrophils release calprotectin into the infection environment during neutrophil extracellular trap (NET) formation as part of NETosis (153). Upon release, calprotectin bound with calcium readily chelates manganese and zinc, preventing the uptake of these metals by bacteria (149–153). Calprotectin also serves as a damage-associated molecular pattern (DAMP) by binding to the Toll-like receptor 4 (TLR4) and receptor for AGE (RAGE), which induces a proinflammatory response (101, 151). Individuals with insulin resistance and T2D exhibit increased levels of circulating calprotectin, further exacerbating the development of T2D (149). Despite elevated circulating calprotectin concentrations in T2D, a recent work from Akbari et al. demonstrates that calprotectin does not sufficiently control GBS infection in diabetic wounds (101). Furthermore, although several bacterial pathogens encode manganese-dependent glycolytic enzymes and superoxide dismutase proteins, some also encode enzymes with redundant functions that are not dependent on manganese (154). These findings highlight that although the nutritional composition of the diabetic infection microenvironment favors bacterial growth, other diabetes-associated factors also contribute to the worsened outcomes observed in diabetic patients. Next, we will discuss how diabetic immune suppression contributes to worsened disease.

## DIABETES-ASSOCIATED IMMUNE SUPPRESSION

The contribution of diabetes-associated immune dysfunction to increased infection frequency and severity is becoming increasingly appreciated. Canonically, diabetes is a group of metabolic disorders that ultimately result in the dysregulation of numerous cellular processes, including glucose uptake, glucose and protein synthesis, and signaling pathways involved with cell growth and differentiation (19–21, 28). As such, the physiological impacts of diabetes extend to most systems within the body, including the immune system (2, 15, 23). To generate an effective immune response, various immune cells undergo metabolic reprogramming mediated by signals from other cells or environmental cues (155). However, the systemic effects of diabetes on normal metabolic processes culminate in a state of immune cell dysfunction, leading to the impaired ability to adequately control and clear invading pathogens (2, 15, 23). Multiple areas of study have documented the barriers to the immune response faced during diabetic infection. Here, we will summarize the impact of diabetes on neutrophils, monocytes, and macrophages, which are crucial mediators for controlling bacterial infections.

Upon infection, host mucosal epithelial cells and phagocytes expressing pathogen-associated recognition receptors (PRRs) recognize pathogen-associated molecular patterns (PAMPs) produced by infiltrating bacteria, including LPS, peptidoglycan, and flagellin (156). Recognition of PAMPs by PRRs elicits a cascade of cell signaling events culminating in the secretion of proinflammatory effector molecules, including proinflammatory cytokines, including interleukin-1 (IL-1), interferon-γ, and tumor necrosis factor (TNF) (156, 157). Diabetes is associated with changes in cytokine profiles during infection, but existing literature presents conflicting findings regarding whether cytokine production is lower or higher in individuals with diabetes. For example, diabetic mice infected with *Porphyromonas gingivalis* exhibit elevated TNF, MIP-2, and MCP-1 levels 3 days post-infection compared with non-diabetic controls (158). Similarly, *in vivo* studies by Sherry et al. demonstrate that administering LPS to mice with T2D elicits greater TNF and IL-1β production in PerMφ and is dependent on elevated glucose levels (159, 160). However, Zykova et al. observed decreased TNF and IL-1β production in peritoneal macrophages (PerMφ) from mice with T2D compared with non-diabetic controls following *ex vivo* treatment with LPS and interferon-γ (161). The discrepancy between these studies could be attributed to differences in glucose levels, as Zykova et al. cultured PerMφ from non-diabetic and diabetic mice in media containing a concentration of glucose lower than the serum levels in non-diabetic mice (161). Nevertheless, Geerlings et al. found that peripheral blood monocytes from individuals with diabetes display reduced production of proinflammatory cytokines following *ex vivo* stimulation with LPS, aligning with the work of Zykova et al. (162). Altogether, the conflicting findings of these studies indicate that alterations in cytokine production in individuals with diabetes may differ based on the degree of diabetes management, underscoring the need for further research.

Diabetes also compromises the ability of innate immune cells to respond to infection through migratory and chemotactic mechanisms (45–47). Neutrophils are the first responders and primary mediators of clearing invading bacteria, rapidly migrating to sites of tissue damage and infection (163). During recruitment to infection sites, circulating neutrophils migrate through the endothelial layer of blood vessels into infected tissue (163). Increased concentrations of TNF within the infection site promote immune cell migration by inducing vasodilation and vascular hyperpermeability (164). However, diabetes is associated with many factors contributing to vascular damage, including endothelial cell damage resulting from oxidative stress, dyslipidemia, and impaired angiogenesis and vasodilation, cumulatively reducing the ability of immune cells to migrate to sites of infection (165). After migrating from the endothelium, neutrophils are recruited to sites of tissue damage and infection by following increasing chemical gradients in a process known as chemotaxis (163). Several studies demonstrate that neutrophils from patients with diabetes display reduced chemotaxis compared with non-diabetic individuals (45–47). Nevertheless, phagocytes localized to diabetic infection sites exhibit a marked reduction in the ability to phagocytose and degrade pathogens (166–170).

Phagocytes utilize numerous bactericidal mechanisms to combat infiltrating microbes, including internalization and subsequent degradation of bacteria during the process of phagocytosis (171–173). Following pathogen internalization, phagocytes rapidly synthesize free radicals to kill phagocytosed pathogens, including reactive oxygen species and NO• (171). Activated phagocytes must first undergo dramatic metabolic reprogramming to generate free radicals (60). Interestingly, the metabolic reprogramming of activated phagocytes is nearly identical to the rewritten metabolic processes observed in cancer cells, known as “aerobic glycolysis” or the Warburg Effect (174, 175). During aerobic glycolysis, glucose is rapidly fluxed through glycolysis and the pentose phosphate pathway to rapidly generate NADPH, which is crucial for superoxide and NO• synthesis (176–178). NADPH oxidase 2, a membrane-bound enzyme on phagocytic vacuoles, generates superoxide by transferring electrons from NADPH to oxygen within the phagosome (173, 179). Moreover, inducible nitric oxide synthase utilizes NADPH as a reducing equivalent to facilitate the oxidation of arginine to NO• (180). Several studies demonstrate that activated neutrophils isolated from both humans and mice with diabetes exhibit a diminished respiratory burst and lower bactericidal activity than neutrophils from non-diabetic counterparts (45, 48, 166–170). In line with these observations, neutrophils, monocytes, and macrophages from individuals with diabetes display deficiencies in generating free radicals (48). The impaired ability of diabetic phagocytes to generate a respiratory burst is seemingly counterintuitive, as there are elevated glucose levels in the diabetic infection microenvironment (58). Part of the reduced phagocytic capacity displayed by neutrophils and macrophages can be attributed to deficiencies in complement activation that result in decreased opsonization and phagocytosis of bacteria (181, 182). However, these results do not fully explain the mechanisms underlying the diminished ability of professional phagocytes to generate free radicals in glucose-replete conditions, suggesting additional mechanisms contribute to this phenotype.

Diabetes may cause dysregulation of phagocyte glucose uptake mechanisms, which serves as a potential explanation as to why phagocytes exhibit impaired free radical generation in glucose-replete conditions (58). Phagocytes depend on rapid glucose uptake to generate a respiratory burst, which is mediated by several high-affinity glucose transporters, including glucose transporter 1 and 3 (GLUT1/3) (183). Neutrophils and macrophages depend on glucose for proper antimicrobial function, which GLUT1 primarily mediates in phagocytes (184). A recent study demonstrated that the burden of *S. aureus* is almost times higher in mice that do not express GLUT1 in phagocytes, and GLUT1 is not expressed in a diabetic SSTI model (58). Another potential explanation is that oxidative stress interferes with normal immune cell function by eliciting improper responses to external stimuli. Neutrophils trap infiltrating bacteria within NETs, which are generated following neutrophil degranulation and the release of intertwined strands of DNA decorated with antimicrobial proteins (185). However, neutrophils circulating in hyperglycemic environments are in a heightened state of oxidative stress and undergo spontaneous apoptosis and NETosis, resulting in increased proinflammatory cytokine production that can exacerbate diabetes severity (153, 186, 187). The most likely explanation for innate immune cell dysfunction in the context of diabetes is due to a combination of factors contributing to dysregulated cellular processes. Despite the current plethora of knowledge regarding the impacts of diabetes on the immune system, the interplay of the diabetic immune response and pathogens is still a burgeoning field.

## THE CONTRIBUTION OF DIABETES TO THE SPREAD OF ANTIBIOTIC RESISTANCE

Increased nutrient availability in the diabetic infection microenvironment and diabetes-associated immune suppression contribute to the increased incidence and severity of bacterial infections in individuals with diabetes (4–6, 24–26). As such, there is a concomitant increase in antibiotic usage in the diabetic patient population (188, 189). An analysis of antibiotic prescription rates between 1995 and 2003 reveals that individuals with diabetes experienced a 60% increase in antibiotic prescriptions for lower respiratory tract infections and a 15% increase for UTIs in individuals with diabetes over time (188). Furthermore, the increase in antibiotic usage during this period corresponded with an increased incidence of infection with MDR bacteria (190, 191). Individuals with T2D are more likely to experience MDR UTIs and respiratory infections (191). MDR pathogens from diabetic UTIs include extended-spectrum β-lactamase-positive Enterobacteriaceae, fluoroquinolone-resistant uropathogens, carbapenem-resistant Enterobacteriaceae, and VRE (192, 193). Additionally, MDR bacteria are becoming more prevalent in DFIs, further exacerbating the difficulty of treating complex DFIs (190). *S. aureus* is the most frequently isolated pathogen from diabetic SSTIs, and recent studies demonstrate that diabetic SSTIs frequently harbor MRSA (194). Other diabetes-related comorbidities that are associated with an increased risk of MDR infection include deep and recurrent ulcers, previous hospitalizations, chronically elevated glycated hemoglobin (HbA1c) levels, neuropathy, and retinopathy (195, 196).

The diabetic infection microenvironment, combined with antibiotic usage, can drive the emergence of antibiotic tolerance and resistance traits in bacterial pathogens (52). Vancomycin is one of the primary frontline antibiotics for treating MRSA infections and has been described as the antibiotic of last resort for treating MDR *S. aureus* (197). The risk of infection with vancomycin intermediate-resistance *S. aureus* (VISA) strains is three times higher in patients with diabetes (52). VISA isolates share several common phenotypes, including thickened cell walls, reduced cell wall autolytic activity, slower growth rates, and decreased virulence (198). The development of intermediate vancomycin resistance in *S. aureus* results from an accumulation of mutations that render *S. aureus* less susceptible to vancomycin (55). Although the molecular mechanisms responsible for VISA development are not entirely defined, there is increased mutation frequency in several genes encoding the WalKR, GraS, and VraSR TCS in VISA isolates, and these genes have therefore been implicated as crucial mediators of VISA strain emergence (197). Selective pressures, or the lack thereof, within the diabetic infection microenvironment may also promote the evolution of VISAs (55). As diabetic tissues contain an abundance of glucose for *S. aureus* to consume with little immune pressure, *S. aureus* can freely accumulate mutations that may ultimately result in the emergence of VISA strains (55, 198).

Concomitant to the emergence of VISA strains in diabetic infections is the evolution of vancomycin-resistant *S. aureus* (VRSA). While VISAs are less susceptible to vancomycin treatment, VRSA strains are fully resistant to clinically achievable vancomycin concentrations (55). Unlike VISAs, where numerous mutations can induce tolerance and a slight increase in the minimum inhibitory concentration to vancomycin, VRSA strain emergence occurs via horizontal gene transfer of the *vanA* operon on the mobile genetic element Tn*1546* originating from VRE into vancomycin-sensitive *S. aureus* (VSSA) strains (98–100). Successful horizontal gene transfer of Tn*1546* from VRE to VSSA necessitated co-localization of VSSA and VRE (98). *Enterococcus* spp. and *S. aureus* frequently co-infect diabetic SSTIs, and coincidentally, one of the first VRSA strains in the United States was isolated from a diabetic patient in 2002 (98, 199). Furthermore, most VRSA strains to date have been isolated from diabetic wounds of patients co-infected by VRE and VRSA (55). From 2002 to 2022, there have been a total of 16 confirmed VRSA infections in the United States, with 12 of these cases occurring in individuals with diagnosed diabetes (54). These results suggest that polymicrobial communities within the diabetic infection environment may serve as a reservoir for novel antimicrobial-resistant bacterial strains.

Diabetes-associated immune suppression may also contribute to the emergence of antimicrobial-resistant bacterial strains. A study by Hou et al. describes the acquisition of antibiotic resistance traits in *Acinetobacter baumannii* using a sequential lung infection model (200). In this study, *A. baumannii* was serially passaged in the lungs of neutropenic and immunocompetent mice treated with or without the antibiotic ciprofloxacin. Results showed that the pulmonary burden of *A. baumannii* propagated in ciprofloxacin-treated neutropenic increased 100-fold by the final passage compared with the initial passage. Conversely, no increase in bacterial burden was observed in *A. baumannii* propagated in immunocompetent mice. Hou et al. attributed the results of these studies to the selection of *A. baumannii* variants with altered ciprofloxacin sensitivity profiles, which primarily arose in neutropenic mice. In line with the findings of Huo et al., longitudinal clinical data from patients receiving long-term vancomycin treatment show that clonal VISA isolates from the same patient exhibit an increased tolerance to vancomycin (201, 202). Both VISA and VRSA isolates are significantly less virulent than VSSA strains (55, 197). However, previous work indicates that low-virulence *S. aureus* strains are still capable of causing invasive infection in individuals with diabetes (86), suggesting that the altered nutrient composition of diabetic tissues, diabetic immune suppression, or even both of these factors may allow for unchecked growth of bacteria with attenuated viability.

## CONCLUDING REMARKS

As the global incidence of diabetes continues to rise, it is imperative to understand the multitude of health complications associated with this group of metabolic disorders (1). Individuals with diabetes are more susceptible to contracting bacterial infections and often exhibit worsened infection outcomes compared to non-diabetic individuals (2–7). The mechanisms underscoring the increased incidence and severity of bacterial infections in individuals with diabetes are multifaceted. In this review, we discuss how the altered nutritional composition of diabetic tissues, diabetic immune suppression, and antibiotic resistance contribute to the increased frequency and severity of bacterial infections.

Chronic hyperglycemia is a strong predictor for increased infection rates among individuals with diabetes (43–47). Numerous bacterial pathogens preferentially utilize glucose as a carbon source (4–6, 24–26). Glucose fuels bacterial growth and virulence and further compounds the difficulty of treating multispecies infections by enhancing biofilm formation, culminating in prolonged and invasive infections (29, 35, 38). Diabetes is further associated with compromised innate immune cell functions, from migrating to infection sites to employing bactericidal mechanisms to destroy invading pathogens (45–48). During phagocytosis, phagocytes utilize increased glucose consumption rates to generate bactericidal free radicals (172, 173). However, the metabolic dysregulation associated with diabetes results in lower glucose consumption and impaired free radical production, resulting in ineffective bacterial killing (58). Finally, diabetes is associated with decreased antibiotic therapeutic efficacy despite higher rates of antibiotic usage (190, 191). Furthermore, individuals with diabetes exhibit an increased incidence of infections with MDR bacteria, and the diabetic infection microenvironment may serve as a reservoir for emergent antibiotic-resistant bacterial strains (55, 98, 188, 189, 199).

The combination of altered nutrient availability in the diabetic infection microenvironment, alongside deficiencies in the innate immune response and the prevalence of altered antibiotic tolerance and resistance profiles in diabetes-associated infections, all contribute to worsened clinical outcomes in individuals with diabetes. Numerous factors outside of those discussed in this review further contribute to worsened disease states in individuals with diabetes. This review aimed to provide a brief overview of three prominent threats contributing to worsened disease in individuals with diabetes.
